# Refining the definitions of cultural safety, cultural competency and Indigenous health: lessons from Aotearoa New Zealand

**DOI:** 10.1186/s12939-025-02478-3

**Published:** 2025-05-09

**Authors:** Elana Curtis, Belinda Loring, Rhys Jones, David Tipene-Leach, Curtis Walker, Sarah-Jane Paine, Papaarangi Reid

**Affiliations:** 1 Faculty of Medical and Health Sciences, Te Kupenga Hauora Māori, University of Auckland, Auckland, New Zealand; 2 Te Kura I Awarua Rangahau Māori Research Centre, Eastern Institute of Technology, Hawke’s Bay, New Zealand; 3 Te Pae Hauora O Ruahine O Tararua, Te Whatu Ora MidCentral, Palmerston North, New Zealand

**Keywords:** Cultural safety, Cultural competency, Indigenous, Māori, Disparities, Inequity, Ethnic

## Abstract

Eliminating Indigenous and ethnic health inequities requires culturally-competent and culturally-safe health workforces and systems. Health professional training institutions and regulatory bodies are increasingly including cultural competency and cultural safety in health professional accreditation standards, and pre-service and in-service training programmes. However, there are mixed definitions and understandings of cultural competency and cultural safety, and how best to achieve them. In 2019, we published a review of international understandings of these terms, and proposed an Indigenous-led definition for cultural safety that we believed to be more fit for purpose in achieving health equity. We also clarified essential principles and practical steps to operationalise this approach in healthcare organisations and workforce development. The aim of this paper is to share our expert reflections upon the experience over the six years since 2019, of implementing this definition in an Aotearoa New Zealand (NZ) context. Recent work undertaken with health regulatory bodies in NZ to refine the understandings of cultural competency, cultural safety and Indigenous health has extended our positioning on these important concepts. A practical example of how these related but distinct concepts apply to Indigenous health is presented.

## Introduction

Achieving health equity should be among the ultimate purposes of any just health system [1–3]. Eliminating health inequities existing along cultural dimensions, including ethnicity and Indigenous status [4], requires culturally-competent and culturally-safe health workforces and systems [5, 6]. Jurisdictions in countries including Australia, Aotearoa New Zealand (NZ), Canada and the United States of America are increasingly including cultural competency and cultural safety in their expectations for health services [7], health professional accreditation standards [8–10], and pre-service and in-service training programmes [11, 12]. However, between and within countries there are mixed definitions and understandings of cultural competency and cultural safety, and how best to teach, assess and achieve them [13–16]. As the authors of a paper defining cultural safety published in the *International Journal for Equity in Health* in 2019 [13], we present our own collective expert reflections on its implementation in NZ and propose further refinements to support the clearer communication and advancement of cultural safety, cultural competency and the related but distinct commitment required for Indigenous health.

## Authorship expertise

As the original authors of 2019 definition paper, we collectively reflect expertise that includes:

- Indigenous/Kaupapa Māori positioning
- Health professional (clinical and health systems) expertise
- Medical education expertise, including accreditation
- Expertise in teaching and assessing cultural safety, cultural competency and Indigenous health
- Expertise in conceptualisation, research, monitoring and interventions for health equity.

Collectively, our experience has been at the forefront of cultural competency since the 1980s, and subsequent cultural safety development in the medical profession in NZ, Australia and internationally.

### Recap of 2019 cultural safety definition

In 2019, we published a review of international understandings of key terms associated with cultural competency and cultural safety, and we then proposed an Indigenous-led definition for cultural safety for the medical profession in NZ that we believed to be more fit for purpose in achieving health equity [13]. We noted that cultural competency, was most often individualised rather than contextualised to organisational/systemic processes, and was focussed on the acquisition of cultural-knowledge. There was no element of the reflective self-assessment of the power, privilege and biases of medical practitioners that is in fact the core focus of cultural safety. We considered at the time that while there was still a need for all health professionals to have a degree of knowledge and understanding of other cultures, an approach to cultural competency that focused purely on acquiring knowledge, skills and attitudes about the ‘exotic other’ was problematic because it supposed that competency, which exists to support equitable outcomes, can be fully achieved [17] by centering the focus on learning about other cultures. We were concerned that an uncritical promotion of cultural competency could lead to increased cultural stereotyping^1^ and cultural essentialism^2^ (both of which should be avoided). We therefore argued for a shift towards the more transformative positioning of cultural safety, involving an ongoing critique of power imbalances and critical self-reflection of the implications for professional practice. We recommended the following definition for cultural safety:

“Cultural safety requires healthcare professionals and their associated healthcare organisations to examine themselves and the potential impact of their own culture on clinical interactions and healthcare service delivery. This requires individual healthcare professionals and healthcare organisations to acknowledge and address their own biases, attitudes, assumptions, stereotypes, prejudices, structures and characteristics that may affect the quality of care provided. In doing so, cultural safety encompasses a critical consciousness where healthcare professionals and healthcare organisations engage in ongoing self-reflection and self-awareness and hold themselves accountable for providing culturally safe care, as defined by the patient and their communities, and as measured through progress towards achieving health equity. Cultural safety requires healthcare professionals and their associated healthcare organisations to influence healthcare to reduce bias and achieve equity within the workforce and working environment”.

### Lessons from implementation of 2019 definition

Since 2019, our definition of cultural safety has seen increasing traction across Australia and NZ, including by the Medical Council of New Zealand (MCNZ) [9], the Council of Medical Colleges [18], other health professional regulatory bodies [19, 20], and government agencies [21]. There has been leadership from the Australian Medical Council that accredits medical schools in Australia and NZ, as well as vocational medical specialist colleges in Australia and those that are bi-national, including the Royal Australasian College of Physicians [22], and the Australian and New Zealand College of Anaesthetists [23].

While this uptake has been encouraging, we have observed as members of our communities, and through our work as educators, policy advisors, researchers and health practitioners, several common phenomena which have given us cause to further refine the way we define and communicate the concepts of cultural safety and cultural competency:

1. While a commitment to cultural safety may be expressed clearly, the understanding is frequently superficial and/or confused [24] – for example, the content of most existing cultural safety training is actually more aligned to cultural competency rather than critical consciousness.
2. On the flip side, we notice that cultural competency has tended to be dropped in favour of cultural safety, rather than continued as a distinct domain (for example, the MCNZ replacing its Statement on Cultural Competence with a Statement on Cultural Safety). Whilst we acknowledge that there is overlap between these two concepts, we are proposing a clear distinction between the two, in order to ensure that the critical competencies within cultural safety and competency are understood and achieved by the health sector.
3. Frequently, cultural safety has been enmeshed with Indigenous health, which blurs obligations for both, and fails to acknowledge the relevance of cultural safety and cultural competency for people of all cultures and identities.
4. Tools and interventions for training and developing cultural safety, particularly its measurement/assessment, are still underdeveloped, and mostly focus on individual self-reflection, self-assessment and weak process measures only [25]. There is growing demand for “practical how-to” guidance beyond merely conceptual understanding, which is currently unmet by the existing resources.
5. Cultural safety remains under-theorised and contested. In particular, there is tension between behavioural interventionist theories, which focus on addressing inequities through individual behaviour change [26] (eg “unconscious bias” training), and critical race theorists, which view structural change as essential to address the institutional, systemic, and individual drivers of privilege and oppression [27]. Further flavouring this debate is that critical race theories are being actively attacked and rejected by right wing politics [28, 29].
6. Lastly, we note that the organisational responsibility for cultural safety has been less well embraced – for the most part cultural safety is viewed as an individual requirement only.

Reflecting on these observations, we believe there is a need to more clearly differentiate the concepts of cultural safety and cultural competency, and the reasons why both are required. We therefore offer several refinements to the articulation of cultural safety and cultural competency, which we believe help advance the evolving international understanding of these critical concepts, and support efforts to enhance the cultural safety and cultural competency of our health systems and workforces, to achieve health equity for people of all identities and cultures.

In 2024, two NZ health regulatory bodies (the MCNZ and the Dental Council) commissioned some of the authors to undertake a review of their health professional competencies and standards in the domains of cultural safety, cultural competency and Indigenous health. The MCNZ had been the earliest adopter of the 2019 definition of cultural safety, releasing a Statement on Cultural Safety in October 2019 [9] which set out professional standards for doctors. In this statement, the MCNZ note that it replaces the MCNZ’s previous 2006 Statement on Cultural Competence. In 2024, the 2019 Statement became due for review, and the Dental Council was similarly keen to ensure its training accreditation standards, professional competencies and professional standards for oral health professionals were in line with current best practice.

Although our most recent review has been focused on standards for doctors and oral health professionals in NZ, we believe these principles are broadly applicable across other health professions, and to non-regulated healthcare workers, including those in non-clinical roles such as booking clerks, human resources personnel and managers.

### Proposed 2025 definitions of cultural competency and cultural safety

**Fig. 1:**
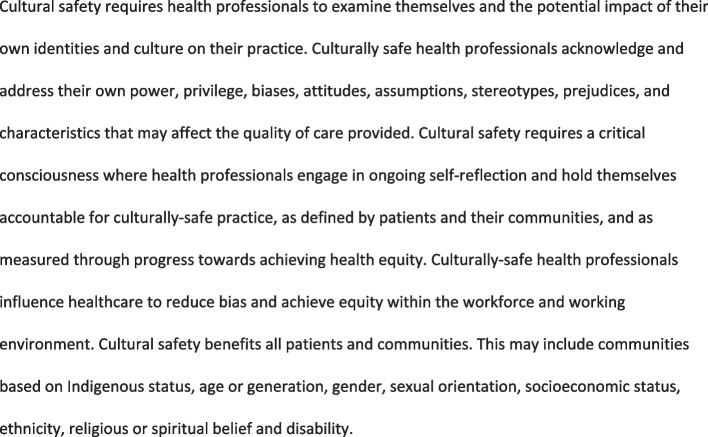
Cultural safety definition

**Fig. 2:**
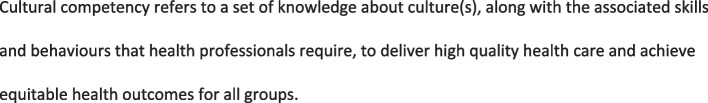
Cultural competency definition

This process of reflection, and review of standards for the MCNZ and DCNZ, has resulted in us proposing clearer definitions of cultural safety (Fig. 1) and cultural competency (Fig. 2) and a clearer differentiation between these concepts and the area of Indigenous health. Cultural safety and cultural competency are related but distinct concepts. Cultural competency and cultural safety are both necessary aspects to achieve Indigenous health, however they are not restricted to Indigenous health.

We understand “culture” in a broad sense to refer to group identities, that can include thoughts, communication, actions, customs, beliefs, values and institutions, of an ethnic/religious/social group [30]. We also note that culture is dynamic, mobile and changes according to time, individuals and groups [31]. Culture is often misunderstood as a characteristic primarily applying to groups other than the dominant social group. When this occurs, the cultural beliefs of those in the dominant group are seen as the unquestioned “norm”. The concept of culture thus extends beyond ethnicity and includes, but is not restricted to: age or generation; gender; sexual orientation; occupation and socioeconomic status; ethnic origin or migrant experience; religious or spiritual belief; and disability. Cultural safety and competency therefore benefit all patients and communities. This may include communities based on Indigenous status, age or generation, gender, sexual orientation, socioeconomic status, ethnicity, religious or spiritual belief and disability [31].

This understanding builds upon and aligns to the core tenets of cultural safety as articulated by Dr Irihapeti Ramsden [32], a Māori nurse practitioner, educator and academic who promoted the term of cultural safety during the 1990s and whose work was instrumental in embedding cultural safety into educational curricula and regulatory standards for nursing and midwifery in NZ [31]. Ramsden’s principles of cultural safety included the need for professionals to undertake critical self-reflection; minimising power differences; undertaking transformative/decolonial action; and that cultural safety must be determined by the person/community receiving care. This also aligns to the concept of critical consciousness, originally described by Paulo Freire in the 1970 s, which involves the capacity to both critically reflect and act upon the factors maintaining oppressive environments [33]. It is important to remember that cultural safety applies to the safety of people training and delivering care, as well as to those receiving it [32].

### Practical guidance for cultural safety

In practice, we consider the following to be the core aspects of cultural safety. These core aspects incorporate the need for self-reflection, acknowledgement of power differentials and taking transformative action.

1. Recognise and respect diverse understandings of health, cultural practices relating to health, family structures and community supports. Patients and staff of all identities have a right to equitable treatment. The concept of culture extends beyond ethnicity and includes, but is not restricted to, age or generation; gender; sexual orientation; occupation and socioeconomic status; ethnic origin or migrant experience; religious or spiritual belief; and disability.
2. Recognise how various forms of marginalisation and privilege impact health need, health care access, service delivery and quality, and health outcomes. Some dimensions of identity confer power and privilege (e.g. white privilege, cisgender privilege, religious privilege), whereas others may result in oppression. Most people, including health professionals and patients, live with one or more intersecting dimension of marginalisation. Importantly, most people also carry one or more intersecting dimension of social privilege. An individual’s ability to engage in their health care may be limited due to their social resources and health inequities [34]. It is important for health professionals to understand their own identities, cultures and dimensions of power/privilege, and how these potentially impact their practice.
3. Eliminate bias in your practice, including in teaching, management, workforce, research, collection and use of data, and delivery of care. Bias in healthcare exists in multiple dimensions, including in teaching, management, workforce, research, collection and use of data, and delivery of care. Policies, protocols, processes, and structures in a practice environment can reinforce privilege, and maintain disadvantage, or enable change [35]. Health professionals should actively engage in using these levers positively to enhance cultural safety and reduce bias. This includes identifying and addressing their own bias, as well as challenging the bias of individual colleagues or systemic bias within the health system. This requires health professionals to be able to recognise incidents of discrimination and microaggressions by other health professionals, colleagues, patients, or their family members and know what options would be available to address these. This can also include amplifying and adding support to the voices of members of marginalised groups who are reporting experiences of discrimination or unsafety. Health professionals can use their own power and privilege in an active way to be critical allies^3^ to oppressed groups [36].
4. Follow national data protocols for the collection, classification and analysis of ethnicity data, and assess the effectiveness your professional practice by ethnicity. Consistent monitoring of health inequities over time are essential to ensure progress. High quality ethnicity data collection is critical to measuring equity in healthcare. For example, in NZ national protocols exist [37] for how ethnicity data should be collected, classified and analysed in the health sector. The entire healthcare system has an obligation to comply with these protocols, and includes conducting regular audits, and implementing measures to improve ethnicity data quality if required.
5. Show evidence of your own ongoing assessment and critique of your cultural safety and commitment to continuous improvement. Ongoing self-reflection, assessment and learning are important steps to ensure health professionals are culturally-safe. However, cultural safety needs to be defined by patients and their communities, particularly those from marginalised groups [32] – so it is not possible to determine cultural safety through self-reflection alone. To assess their cultural safety, health professionals need to seek out additional sources of feedback, for example, peers, staff and patients, as well as data from their practice (e.g. ethnicity data quality audits, outcomes data, staff satisfaction data). Health professionals need to act upon their self-reflection and external sources of feedback, by undertaking explicit activities to further their cultural safety, and evaluate the effectiveness of these measures.

### Practical guidance for cultural competency

In practice, we consider the following to be the core components of cultural competency. As with cultural safety guidance above, while originally written for individual health professionals, this framework also provides a sound basis for considering the cultural competency of institutions:

1. Understand how culture affects health. Health professionals work within a population that is culturally diverse. Culture is dynamic and changes over time, extends beyond ethnicity, and patients and colleagues may identify with multiple cultural groupings at any one time. Culture affects how both health professionals and patients understand health and illness, how health services are offered and accessed, and the effectiveness of health care interventions. Cultural beliefs of the dominant group are often seen as the unquestioned “norm”. For example in NZ, the health care system, institutions and medical practice are strongly informed by white British colonial culture. For those belonging to the dominant cultural groupings, the systems of society, including health services, are often designed to work well for them (in terms of alignment with beliefs, practices, and understandings). For those belonging to cultures outside the dominant cultural group, health services often do not work as well, because they are designed based on a different set of cultural understandings and norms.
2. Be aware of your own cultural identities and how these potentially impact your practice. Health professionals need to be aware of their own cultural identities, beliefs and worldviews, and how these impact their medical practice. Health professionals need to avoid assuming their worldview applies to others, and not impose their cultural values and practices on patients and colleagues.
3. Recognise which cultural worldviews apply to patients and colleagues, and adjust your practice accordingly to achieve the best health outcomes. It is important that health professionals create a safe environment for their colleagues and patients to share information about their cultural identities and worldviews. This can involve tailoring their practice (including communication style, practice systems and processes) to be appropriate to the cultural identities of patients and colleagues (as defined by them). Health professionals should avoid cultural stereotyping and cultural essentialism. This also requires an understanding of the difference between cultural appreciation^4^ and cultural appropriation^5^ (and the harms associated with appropriation). Health professionals need to be able to work with people in respectful and non-judgmental ways.
4. Evaluate your cultural competency professional development needs on an ongoing basis, and address these needs. Cultural competency does not mean that a health professional can ‘master’ or become fully competent in other cultures. Rather, cultural competency requires health professionals to undertake ongoing self-reflection and adaptation of their practice in order to operate effectively for all cultural groups. Health professionals should use objective measures to assess their cultural competency, identify areas for improvement and monitor their ongoing development of cultural competency. It is important that health professionals identify when they require additional cultural expertise to assist them in delivering culturally-competent care and know where to access this expertise. It is also important that health professionals seek to reduce the unwanted and uncompensated cultural load^6^ on colleagues and patients from non-dominant cultural groupings in their practice.

### Practical advice on how cultural safety and cultural competency relate to Indigenous health

As noted above, cultural competency and cultural safety apply to a broad range of cultural groups, especially where power imbalances/marginalisation are involved, and are certainly not uniquely relevant to Indigenous groups. Additionally, both cultural safety and cultural competency form part of what is needed to address Indigenous health inequities, but they do not capture the full requirements for Indigenous rights (and Indigenous rights extend beyond the right to health) [38].

In addition to cultural safety and cultural competency, these are the key attributes of health professionals (and organisations) required to provide safe and competent care for Indigenous peoples:

1. Understand that Indigenous peoples have Indigenous rights to health (above and beyond any extra health need).
2. Understand that Indigenous peoples often have higher unmet health needs (independent of other factors e.g. socioeconomic status).
3. Have a critical understanding of the determinants of Indigenous health inequities, including the role of health professionals in creating, maintaining or eliminating inequities.
4. Recognise that there are diverse and dynamic ways of being Indigenous and Indigenous peoples live in a variety of geographic, cultural and socioeconomic contexts.
5. Have knowledge of key aspects of traditional and contemporary Indigenous values and cultural practices, and the implications of these for your practice.
6. Assess the equity of your practice for Indigenous peoples, and implement measures to address any inequities you identify.
7. Show evidence of skill development with respect to cultural safety and cultural competency, and demonstrate how you are using this to benefit Indigenous peoples through your practice.

## Synthesis of key points

Through our experience in articulating and embedding cultural safety in NZ, a number of lessons have emerged which offer value to an international audience:

1. Both cultural competency and cultural safety are important. In 2019, we outlined some of the ways that a narrow focus on cultural competency could be harmful and argued for a shift towards a transformative concept of cultural safety, involving a critique of power imbalances and critical self-reflection. “Other-focused” approaches to cultural competency promote oversimplified understandings of other cultures based on cultural stereotypes and focus on acquiring knowledge, skills and attitudes rather than critiquing the causes of health inequities. We noted that health professionals and organisations often pursued cultural competency activities but stopped short of the more challenging and confronting work of critical consciousness and cultural safety. In some of the adoption of our cultural safety definition, there has been a tendency to replace cultural competency with cultural safety (for example, the 2019 MCNZ Statement on Cultural Safety replaced the 2006 Statement on Cultural Competency). This impact was not intentional, and it is our view that both cultural safety and cultural competency are required. Our more recent work with both the MCNZ and Dental Council has sought to provide greater clarity about how these two concepts are related but distinct. To some, this distinction may seem slight or artificial. We acknowledge that the difference we are drawing between cultural safety and cultural competency may seem subtle and there are certainly common elements. For example, both cultural safety and cultural competency require an understanding that cultural identities impact health seeking behaviour, norms and understandings. Both require ongoing assessment of competency and development. However, cultural safety focuses on the analysis of how power and privilege are distributed (among professionals, patients and organisations), and how these factors impact on health care delivery and need. Cultural competency focuses on an awareness of the presence of different cultural beliefs and worldviews (among professionals, patients and organisations), and how to behave appropriately to practice effectively in different cultural contexts.
2. Cultural safety and competency are for all groups. In the NZ setting, cultural safety and cultural competency have most commonly been applied to Māori (Indigenous) health. This has led to confused understanding of health professionals’ requirements for cultural safety/competency more broadly, and their obligations to Indigenous people. We need to clearly disentangle these concepts, to communicate that cultural safety and cultural competency are important for people of all cultures/identities, and that both these concepts are important (but not only these) for Indigenous health. Cultural safety and competency benefit all patients and communities. Cultural competency and cultural safety are both necessary aspects of health systems to achieve Indigenous health, however they are not restricted to Indigenous health, nor do they fully capture Indigenous rights. Indigenous health teaching/training should include all three concepts: cultural competency, cultural safety and Indigenous health—and be taught by Indigenous staff.
3. Cultural safety needs to explicitly focus on power. Fundamental to understandings of cultural safety is the notion of ‘power’ [31, 39–41]. Cultural safety foregrounds power differentials within society, the requirement for health professionals to reflect on interpersonal power differences, and how the transfer of power within multiple contexts can facilitate appropriate care [31]. Whilst this was intrinsic to our understanding of cultural safety, we did not include power explicitly in our 2019 definition, instead recommending that health professionals organisations should “acknowledge and address their own biases, attitudes, assumptions, stereotypes, prejudices, structures and characteristics” [13]. In the 2024 definition, we have made it explicitly clear that this also includes examining power and privilege. Addressing power differentials also requires transferring power to patients and communities to comment on the safety of their healthcare experiences from a cultural perspective.
4. Cultural safety and competency in health applies to more than just clinical encounters. Power differentials and biases in health systems contribute to health inequities via a myriad of ways, not just through individual clinical interactions between a health professional and a patient. In 2024, we have sought to make it clearer that health professionals and organisations need to consider cultural safety and competency in terms of a much broader sense, including workplace policies, staff dynamics and culture, training and infrastructure. We have encouraged regulatory bodies to link cultural safety requirements to the full scope of practice. For example, the MCNZ defines medical practice as “wider than clinical medicine to include teaching, research, medical or health management, in hospitals, clinics, general practices and community and institutional contexts, whether paid or voluntary” [42]. Further to this is that staff (including students and trainees) of all identities also have a right to cultural safety and competency in their own working environment just as a culturally safe working environment is needed to provide safe care for patients, whānau (families) and address health inequities. Cultural safety curricula and standards have so far focussed on individual practitioners. There remains an urgent need to move to thinking beyond individual responsibility, to developing culturally safe and culturally competent organisations.
5. Going beyond “self-reflection” to objective measurement and tools. Because it asks us to turn our critical eye upon ourselves, cultural safety is inherently more challenging to teach and assess than concepts based on knowledge and skills [18]. However, a lack of structured training tools and methods of objective assessment mean that there is a currently risk of relying on “self-reflection”, and self-assessment, alone. In NZ, a Cultural Safety Training Plan for Vocational Medicine in Aotearoa was developed as a guide for the Council of Medical Colleges to assist their member colleges in the development of high quality training and continuous professional development (CPD) packages for doctors [18]. This plan certainly requires ‘self-reflection’ but also has such things as ‘clinical audit by ethnicity’ and a ‘transformative change plan’ included. While there must be an assessment of these in vocational training there is no formal assessment for specialists other than the annual self-recording of CPD activities by each individual doctor. Further developments in the domain of teaching and assessment for cultural safety, must ensure that the same degree of rigour is applied around assessment as is applied to other necessary competencies for health professionals.
6. Cultural safety is politically vulnerable, especially where baseline cultural safety is low. Since we introduced our definition for cultural safety in 2019, we have observed progression in academic thinking on cultural safety and its importance to health practice. However, the broader political receptivity to concepts associated with cultural safety, competency and Indigenous rights to health has regressed within a NZ context overall (e.g. the recent Cabinet Office circular requiring all central government organisations to prioritise public services on “the basis of need not race” [43]). Achieving a mature and meaningful commitment to cultural safety may not be a linear process with cultural safety remaining politically vulnerable. Furthermore, the existing low level of cultural safety and cultural competency among health professionals may be contributing to a circular loop of resistance to including, developing and strengthening these competencies further. We recommend that health professionals and organisations stay vigilant to this vicious feedback loop in order to progress health equity overall.
7. There remains a need to accept multiple understandings. As we noted in 2019, terms such as cultural safety are understood in different ways in different places. Multiple definitions may not be problematic in its own right – although can create challenges when one regulatory body operates across multiple jurisdictions. For example, the Australian Medical Council (AMC) is an independent body which sets standards for medical education and assessment across both Australia and NZ. They note that Australia and NZ both define cultural safety differently [9, 10], and that it is not the purpose of AMC to need those aligned [44]. Even within NZ, cultural safety arises from a different historical origin and positioning for different professions (e.g. nursing compared to medicine in NZ [45]), so defining these concepts will remain an ongoing challenge. There is also some resistance from Indigenous perspectives that adhere to a more essentialist positioning of Indigenous knowledge – which foregrounds traditional Indigenous knowledge as the only solution (for understandable reasons because of the need to reclaim/recentre traditional Indigenous knowledge given the impact of colonisation). However, addressing health inequities requires a structural critique of power and privilege, which requires anti-racist and structural interventions, above and beyond solutions arising from Indigenous knowledge alone.

## Conclusion

We present refinements to our understanding and expression of cultural safety and cultural competency, based on insights and learning from attempting to embed these concepts in a NZ context. We propose some improvements to our 2019 definitions, and believe that a willingness to revisit and critique definitions is a strength. In fact, we consider that a commitment to ongoing critique and continuous improvement or ‘critical consciousness’ is at the heart of cultural safety. Whilst we write from an Indigenous perspective, concepts such as cultural safety are fundamentally emancipatory, and serve to benefit all marginalised groups. We look forward to the continuing refinement and improvement of our collective understanding of these concepts globally, and the continual development arising from their greater application.

## Data Availability

No datasets were generated or analysed during the current study.

## Footnotes

1 Cultural stereotyping means making assumptions about an individual based on their cultural identity.

2 Cultural essentialism involves assuming there is one fixed/right way to belong to a particular cultural category.

3 Critical allyship refers to behaviours people can use to counteract the unfair social systems and power imbalances which create health inequities. Allyship includes: avoiding trying to save or fix people; taking active steps to learn about the systems of inequality for which one is in a position of privilege; creating space/speaking less; recognising the need for action at systemic, institutional, interpersonal and internal levels; and not using allyship to enhance personal power.

4 Cultural appreciation can be described as a way of honouring another culture through exploration and seeking an understanding as a way to honour that culture, beliefs, and traditions.

5 Cultural appropriation refers to the use of elements of one culture by members of another culture, in ways that are perceived as unacknowledged or inappropriate.

6 Cultural load is the (often invisible) additional workload borne by people of certain cultural groups to provide their cultural expertise in a professional setting, on a voluntary or undercompensated basis without any reduction/alteration to their normal workload.
